# Supplementary material for: Where does the carbon go? A new carbon balance method to assess what happens to plastics under solar exposure
Source: PLoS One. 2025 Sep 29;20(9):e0333021. doi: 10.1371/journal.pone.0333021 (PMC12478910; doi:10.1371/journal.pone.0333021)
Supplement: S1 SupInfo — (DOCX) [file pone.0333021.s001.docx]

Supporting information

Where does the carbon go? A new carbon balance method to assess the fate of plastics under solar exposure

Gustave Bertier^a,b^, Arnaud Martel^b,c^, Matthieu George^a^, Pascale Fabre^a^, Fabien Boucher^c^, Justine Gérome, Fabienne Lagarde^b,c^*

a: Laboratoire Charles Coulomb - Université de Montpellier, France

b: Institut des Molécules et Matériaux du Mans - Université du Mans, France

c: IUT du Mans - Université du Mans, France

*: corresponding author


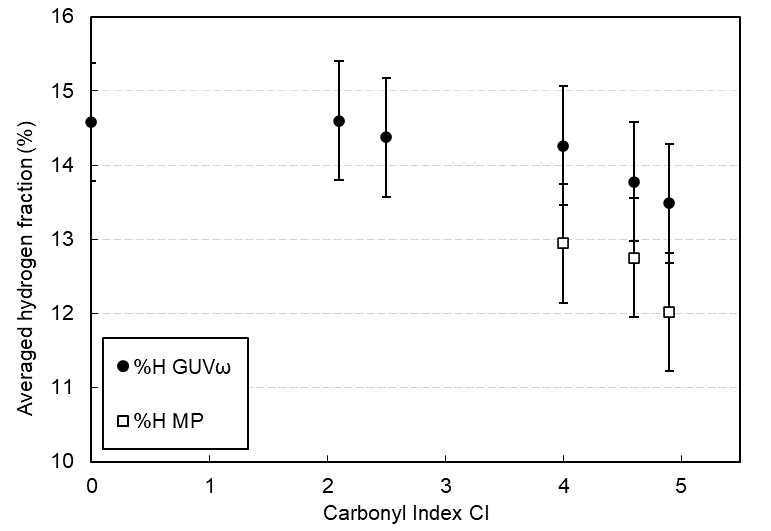


Fig 1: Evolution of the hydrogen content of GUVω and MP in function of the post aging carbonyl index as measured by elemental analysis.


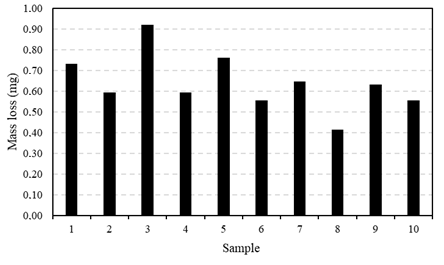


Fig 2: Mass lost by the filter after being used for filtration of ultrapure water. The average value obtained of 0.641mg is used to correct MPs masses


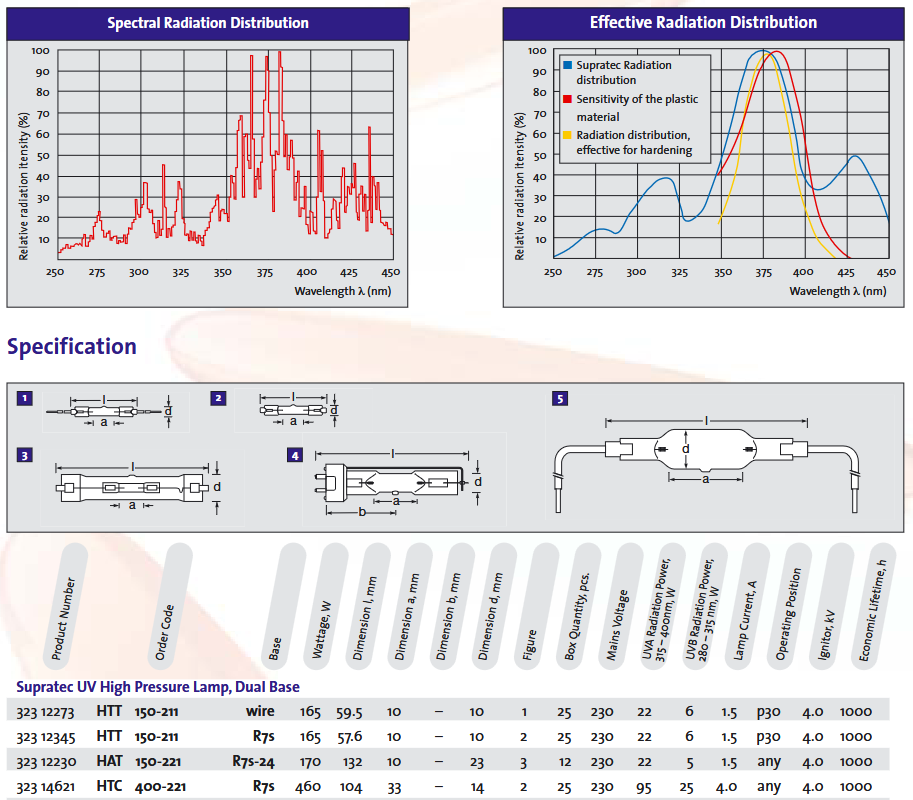


Fig 3: Characteristics of the lamp used. Source: <https://www.sillamps.com/datasheets/Radium.pdf>


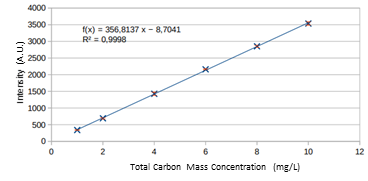


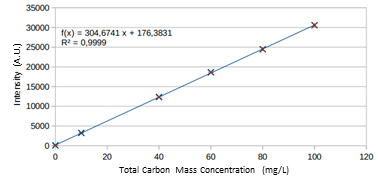


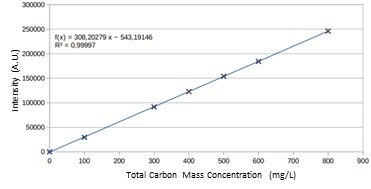


Fig 4: Total Carbon measurement Calibration from 1 to 10, 10 to 100 and 100 to 800mg/L


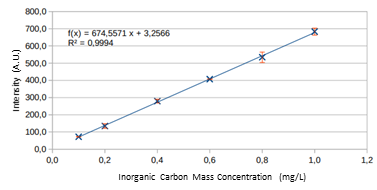


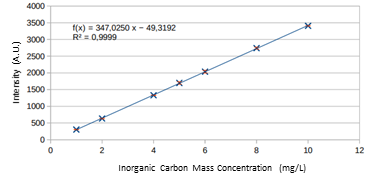


Fig 5: Inorganic Carbon measurement Calibration from 0.1 to 1mg/L and 1 to 10mg/L
